# Cancer incidence in Ghana, 2012: evidence from a population-based cancer registry

**DOI:** 10.1186/1471-2407-14-362

**Published:** 2014-05-23

**Authors:** Dennis O Laryea, Baffour Awuah, Yaw A Amoako, E Osei-Bonsu, Joslin Dogbe, Rita Larsen-Reindorf, Daniel Ansong, Kwasi Yeboah-Awudzi, Joseph K Oppong, Thomas O Konney, Kwame O Boadu, Samuel B Nguah, Nicholas A Titiloye, Nicholas O Frimpong, Fred K Awittor, Iman K Martin

**Affiliations:** 1 Public Health Unit, Komfo Anokye Teaching Hospital, Kumasi, Ghana; 2 Kumasi Cancer Registry, c/o Public Health Unit, Komfo Anokye Teaching Hospital, Kumasi, Ghana; 3 Department of Oncology, Komfo Anokye Teaching Hospital, Kumasi, Ghana; 4 Department of Medicine, Komfo Anokye Teaching Hospital, Kumasi, Ghana; 5 Department of Paediatrics, Komfo Anokye Teaching Hospital, Kumasi, Ghana; 6 Department of Ear, Nose and Throat, Komfo Anokye Teaching Hospital, Kumasi, Ghana; 7 Research and Development Unit, Komfo Anokye Teaching Hospital, Kumasi, Ghana; 8 Metropolitan Directorate of Health Services, Ghana Health Service, Kumasi, Ghana; 9 Department of Surgery, Komfo Anokye Teaching Hospital, Kumasi, Ghana; 10 Department of Obstetrics and Gynaecology, Komfo Anokye Teaching Hospital, Kumasi, Ghana; 11 Kumasi South Regional Hospital, Kumasi, Ghana; 12 Department of Pathology, Komfo Anokye Teaching Hospital, Kumasi, Ghana; 13 Biostatistics Unit, Komfo Anokye Teaching Hospital, Kumasi, Ghana; 14 University of Washington, Northern Pacific Global Health Fellows, Kumasi, Ghana

## Abstract

**Background:**

Data on cancers is a challenge in most developing countries. Population-based cancer registries are also not common in developing countries despite the usefulness of such registries in informing cancer prevention and control programmes. The availability of population-based data on cancers in Africa varies across different countries. In Ghana, data and research on cancer have focussed on specific cancers and have been hospital-based with no reference population. The Kumasi Cancer Registry was established as the first population-based cancer registry in Ghana in 2012 to provide information on cancer cases seen in the city of Kumasi.

**Methods:**

This paper reviews data from the Kumasi Cancer Registry for the year 2012. The reference geographic area for the registry is the city of Kumasi as designated by the 2010 Ghana Population and Housing Census. Data was from all clinical departments of the Komfo Anokye Teaching Hospital, Pathology Laboratory Results, Death Certificates and the Kumasi South Regional Hospital. Data was abstracted and entered into Canreg 5 database. Analysis was conducted using Canreg 5, Microsoft Excel and Epi Info Version 7.1.2.0.

**Results:**

The majority of cancers were recorded among females accounting for 69.6% of all cases. The mean age at diagnosis for all cases was 51.6 years. Among males, the mean age at diagnosis was 48.4 compared with 53.0 years for females. The commonest cancers among males were cancers of the Liver (21.1%), Prostate (13.2%), Lung (5.3%) and Stomach (5.3%). Among females, the commonest cancers were cancers of the Breast (33.9%), Cervix (29.4%), Ovary (11.3%) and Endometrium (4.5%). Histology of the primary tumour was the basis of diagnosis in 74% of cases with clinical and other investigations accounting for 17% and 9% respectively. The estimated cancer incidence Age Adjusted Standardised Rate for males was 10.9/100,000 and 22.4/100, 000 for females.

**Conclusion:**

This first attempt at population-based cancer registration in Ghana indicates that such registries are feasible in resource limited settings as ours. Strengthening Public Health Surveillance and establishing more Population-based Cancer Registries will help improve data quality and national efforts at cancer prevention and control in Ghana.

## Background

Quality data on cancer in developing countries especially sub-Saharan Africa is a challenge for most countries [1]. Cancer registries, which are health units concerned with collecting systematically data on cancers, are useful sources of evidence on cancers. These centres when well established can provide high quality data on cancers as has been advocated for [2] and are useful in planning cancer prevention and control activities [1]. Population-based cancer registries (PBCR) are forms of cancer registries which provide information on cancers in a defined population. PBCRs are useful in estimating the incidence of cancer in specified populations. PBCRs are however not common in Africa and is highlighted by the poor representation of Africa in the global cancer estimates published by the World Health Organisation [3]. There are a handful of PBCRs in Africa with the African Cancer Registry Network (AFCRN) currently championing the cause for the establishment of more PBCRs in Africa [4]. Some nationally-based PBCRs exist in countries like The Gambia [5], city-based ones as the Ibadan and Abuja registries in Nigeria [6] or regional ones as the Eldoret Registry in Kenya [7].

Geographical location [8] or occupational settings [9] have been identified as risk factors for cancer in Ghana. Although smoking is not a significant public health issue in Ghana [10-12] unacceptably high levels of second hand smoking have been found in some places [9]. The recently passed Public Health Act outlaws smoking in public places and is seen as useful in reducing not only the incidence of second-hand smoking but smoking prevalence overall [13]. Human Herpes Virus 8 (HHV 8) associated with Kaposi Sarcoma has also been found to be highly prevalent in Ghana [14] as has Human Papilloma Virus (HPV) infections [15]. Despite the lack of population-based data on cancer in Ghana, there is some evidence of the public health importance of cancer in Ghana [16,17]. The need to develop a comprehensive programme on non-communicable disease control including cancers in Ghana has been highlighted [18,19]. Currently some activities with implications for cancer control and prevention are ongoing in Ghana. Hepatitis B vaccination is an integral part of Ghana’s immunisation programme and may contribute to reducing the incidence of hepatitis B and possibly, liver cancer. Screening for specific cancers such as cervical cancer, although available, has been found to be low [18] while some attempts have also been made to introduce other methods of screening for some cancers hitherto not available in Ghana [20].

The burden of cancer in Ghana has not been static. Several studies on cancers in Ghana have focussed largely on cancers of specific sites [21-23] including the stages of presentation and have mainly been institutionally-based with no reference population [16,17,24,25]. There is the need for more comprehensive studies focussing on populations [26] in order to provide accurate information on cancers for action [2,27]. Some attempts have been made at collecting population-based cancer data in Ghana [28]. Cancer registration particularly population-based ones remain rare in Ghana. The Kumasi Cancer Registry was established in 2012 with the objective of providing population-based data on cancers in Kumasi. We set out to describe cancer cases seen in Kumasi and to estimate incidence using data from the Kumasi Cancer Registry in 2012.

## Methods

This paper reviews data from the Kumasi Cancer Registry (KsCR) for 2012. The KsCR is a member of the AFCRN and started as a hospital-based cancer registry (HBCR) in 2004. It was converted to a PBCR in 2012 with the initial aim of providing data on cancers in the population of Kumasi and subsequently, the Ashanti Region of Ghana. The reference geographic area for the registry is the city of Kumasi as designated by the 2010 Ghana Population and Housing Census.

We reviewed data collected from all clinical departments and the Pathology Department of the Komfo Anokye Teaching Hospital (KATH), Private Laboratory Results, and Death Certificates for 2012. We also reviewed cases recorded at the Kumasi South Regional Hospital. Other sources of data for cancer cases in Kumasi were Pathology Laboratory Reports, Biostatistics Index Cards, Out-patient Records and Haematology Laboratory Records. All cases of cancer were first identified and selected. Cases specific to Kumasi based on the place of usual residence were identified and selected for inclusion in our dataset. Required information including demographic, tumour and other clinical information were collected. Abstracted data was verified by a clinician before entry into CanReg 5 database. Further verification of data quality was conducted by the Registry Manager before confirmation into the database. The International Classification of Diseases for Oncology (ICD-O3) was used for classification and coding of cases of cancers recorded [29].

The incidence of multiple registrations was controlled by the use of multiple variables including the National Health Insurance Number, Date of Birth, Hospital ID number and Age of patient. This was necessitated by the lack of a single form of identification in Ghana. Names were not used as recommended [1] because of the similarity of names as well as variations in the spellings of some names.

Data was exported from Canreg 5 into Microsoft Excel® and analysed using Epi Info Version® 7.1.2.0. Epi Info was used to generate means, frequencies and proportions. Microsoft Excel was used to generate charts and graphs. Canreg 5 was used to generate the crude and ASRs for the cases recorded.

Approval for the use of data from the Registry for the purpose of this publication was obtained from the Kumasi Cancer Registry Advisory Board. Ethical approval was from the Komfo Anokye Teaching Hospital/Kwame Nkrumah University of Science and Technology Committee on Human Research and Publication Ethics.

## Results

The majority of cancers recorded for the period were among females and they accounted for 69.6% of all cases. The basic demographic information of cancer cases recorded in Kumasi for 2012 is as shown in Table 1.

**Table 1:** Basic demographic information of cancer cases in Kumasi, 2012

**Parameter**	**Frequency**	**Percentage**
Sex		
Male	76	30.4
Female	177	69.6
Age group		
<20	15	5.9
20–29	15	5.9
30–39	27	10.7
40–49	52	20.6
50–59	53	20.9
60–69	42	16.6
70–79	37	14.6
>79	12	4.7
Occupation		
Trading	97	38.3
Unemployed	44	17.3
Farming	27	10.7
Teaching	11	4.4
Student	10	4.0
Others	64	25.3

The mean age (SD) at diagnosis for all cases was 51.6 (18.7) years with a median age of 53 years and a range of 1 to 90 years. Among males, the mean age (SD) at diagnosis was 48.4 (17.9) years, median age 48 years and a range of 1 to 90 years. Among females the mean age at diagnosis was 53.0 (18.8) years, median age 54 years and a range of 1 to 90 years.

The commonest sites for cancers reported among both sexes were Breast (24.1%), Cervix Uteri (20.6%), Ovary (7.9%), Liver (6.4%) and Prostate (4.0%).

**Figure 1:**
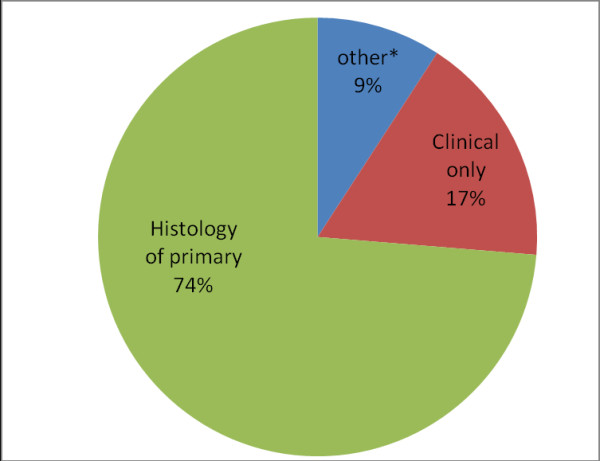
**Basis of diagnosis for cancer cases in Kumasi, 2012.** *includes haematological investigation, ultrasonography and tumour markers.

The most common cancers among males were cancers of the Liver (21.1%), Prostate (13.2%), Lung (5.3%) and Stomach (5.3%). Among females, the commonest cancers were cancers of the Breast (33.9%), Cervix (29.4%), Ovary (11.3%) and Endometrium (4.5%).The histology of the primary tumour formed the basis of diagnosis in most (73.71%) of cases. Figure 1 shows the basis of diagnosis for all cancers in this study.

Among cases histologically diagnosed, grading information was not available for 62 of the cases. For the 123 cases with tumour grading information available, moderately differentiated cases were the leading finding accounting for 49.6% of cases. The proportions for the various grades are as shown in Table 2.

**Table 2:** Tumour types by grade

**Grade**	**Frequency**	**Percentage**
Well differentiated	17	13.8
Moderately differentiated	61	49.6
Poorly differentiated	35	28.5
Undifferentiated	10	8.1
Total	123	100.00

**Figure 2:**
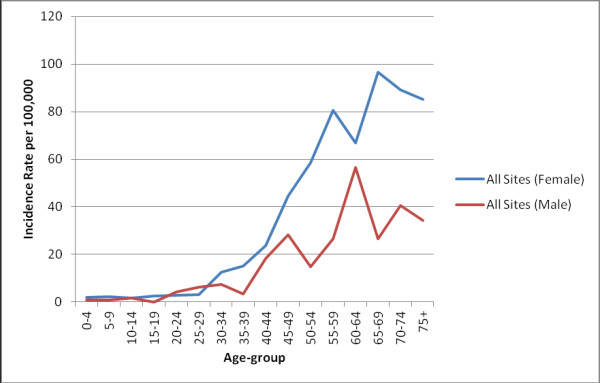
Age specific rates (ASR) for all cancers by sex in Kumasi, 2012.

Based on the current population of Kumasi, the estimated crude cancer incidence for 2012 was 11.9 per 100,000. Among males, the cancer incidence is 7.3/100,000 and 15.7/100,000 among females. The age-standardised incidence rates (ASR) for males was 10.9/100,000 and 22.4/100,000 for females. The ASR for the various tumour sites reported in Kumasi for 2012 is as shown Tables 3 and 4. ASR by sex across all age groups is as shown in Figure 2.

**Table 3:** Frequency, age-specific incidence rates, average annual crude incidence rates, and ASR by site in females in Kumasi, 2012

**Site**	**All ages**	**0-4**	**5-9**	**10-14**	**15-19**	**20-24**	**25-29**	**30-34**	**35-39**	**40-44**	**45-49**	**50-54**	**55-59**	**60-64**	**65-69**	**70-74**	**75+**	**Crude rate**	**ASR**
Salivary glands	2	-	-	-	-	-	-	-	-	-	-	2.5	-	-	-	-	3.9	0.2	0.2
Stomach	1	-	-	-	-	-	-	-	-	-	-	2.5	-	-	-	-	-	0.1	0.1
Colon	1	-	-	-	-	-	-	-	-	-	-	-	-	-	-	-	3.9	0.1	0.1
Anus	1	-	-	-	-	-	-	-	-	-	-	-	-	4.5	-	-	-	0.1	0.2
Gall bladder	1	-	-	-	-	-	-	-	-	-	2.3	-	-	-	-	-	-	0.1	0.1
Nose, Sinuses etc.	2	-	-	-	-	-	-	-	-	1.8	-	-	4.2	-	-	-	-	0.2	0.3
Bone	1	-	-	0.8	-	-	-	-	-	-	-	-	-	-	-	-	-	0.1	0.1
Melanoma of skin	1	-	-	-	-	-	-	-	-	-	2.3	-	-	-	-	-	-	0.1	0.1
Other skin	1	-	-	-	-	-	-	-	-	-	-	-	-	4.5	-	-	-	0.1	0.2
Kaposi Sarcoma	1	-	-	-	-	-	-	1.5	-	-	-	-	-	-	-	-	-	0.1	0.1
Connective and soft tissue	3	-	0.7	-	0.9	-	-	1.3	-	-	-	-	-	-	-	-	-	0.3	0.2
Breast	59	-	-	-	-	-	2.0	5.1	7.6	10.9	21.1	20.4	29.7	31.2	29.7	22.3	11.6	5.3	7.9
Vulva	2	-	-	-	-	-	-	-	-	-	-	-	-	-	-	5.6	3.9	0.2	0.2
Cervix uteri	52	-	-	-	-	-	-	3.8	1.5	7.3	14.1	15.3	25.5	13.4	44.6	33.5	42.7	4.6	6.8
Corpus uteri	8	-	-	-	-	-	-	-	1.5	-	-	5.1	4.2	4.5	7.4	11.2	-	0.7	1.1
Uterus unspecified	1	-	-	-	-	-	-	-	-	-	2.3	-	-	-	-	-	-	0.1	0.1
Ovary	20	-	-	-	0.9	1.8	-	1.3	-	-	2.3	10.2	12.7	8.9	14.9	11.	7.8	1.8	2.6
Placenta	3	-	-	-	-	-	1.0	1.3	1.5	-	-	-	-	-	-	-	-	0.3	0.2
Kidney	2	1.3	-	-	-	-	-	-	-	-	-	-	-	-	-	-	-	0.2	0.2
Bladder	2	2	-	-	-	-	-	-	-	-	-	-	-	-	-	5.6	3.9	0.2	0.2
Eye	1	-	-	-	0.9	-	-	-	-	-	-	-	-	-	-	-	-	0.1	0.1
Brain, nervous system	2	-	-	-	-														
Non-Hodgkin lymphoma	8	0.7	1.5	0.8	-	0.9	-	-	-	1.8	-	2.5	-	-	-	-	3.9	0.7	0.7
Multiple myeloma	1	-	-	-	-	-	-	-	-	-	-	-	-	-	-	-	3.9	0.1	0.1
Other unspecified	1	-	-	-	-	-	-	-	-	-	-	-	4.2	-	-	-	-	0.1	0.2
All Sites	177	2.0	2.2	1.6	2.6	2.8	3.0	12.7	15.1	23.7	44.6	58.6	80.7	66.9	96.7	89.2	85.3	15.8	**22.3**

**Table 4:** Frequency, age-specific incidence rates, average annual crude incidence rates, and ASR by site in males in Kumasi, 2012

**Site**	**All ages**	**0-4**	**5-9**	**10-14**	**15-19**	**20-24**	**25-29**	**30-34**	**35-39**	**40-44**	**45-49**	**50-54**	**55-59**	**60-64**	**65-69**	**70-74**	**75+**	**Crude rate**	**ASR**
Mouth	1	-	-	-	-	-	-	-	-	-	-	-	-	5.1	-	-	-	0.1	0.2
Salivary glands	2	-	-	-	-	-	-	-	1.7	-	-	3.0	-	-	-	-	-	0.2	0.3
Tonsil	1	-	-	-	-	-	-	-	-	-	-	-	4.4	-	-	-	-	0.1	0.2
Oesophagus	1	-	-	-	-	-	-	-	-	-	2.6	-	-	-	-	-	-	0.1	0.2
Stomach	4	-	-	-	-	-	-	-	-	-	2.6	3.0	4.4	5.1	-	-	-	0.4	0.7
Colon	1	-	-	-	-	-	-	-	-	-	-	-	-	-	8.9	-	-	0.1	0.3
Rectum	1	-	-	-	-	-	-	-	-	-	-	-	-	-	-	8.1	-	0.1	0.2
Liver	16	0.7	-	-	-	1.1	2.5	1.5	-	12.2	5.1	-	-	-	8.9	8.1	5.7	1.6	2.0
Gall bladder	1	-	-	-	-	-	-	-	-	-	2.6	-	-	-	-	-	-	0.1	0.2
Larynx	1	-	-	-	-	-	-	-	-	-	-	-	4.4	-	-	-	-	0.1	0.2
Trachea, bronchus and lungs	3	-	-	-	-	-	-	-	-	-	-	-	-	15.4	-	-	-	0.3	0.6
Bone	2	-	-	-	-	1.1	-	-	-	-	2.6	-	-	-	-	-	-	0.2	0.2
Other skin	1	-	-	-	-	-	1.3	-	-	-	-	-	-	-	-	-	-	0.1	0.1
Mesothelioma	1	-	-	-	-	-	-	-	-	-	-	-	-	-	-	-	5.7	0.1	0.1
Kaposi Sarcoma	1	-	-	-	-	-	-	-	-	-	-	3.0	-	-	-	-	-	0.1	0.1
Connective and soft tissue	4	-	-	-	-	-	-	3.0	1.7	-	-	-	4.4	-	-	-	-	0.4	0.5
Breast	1	-	-	-	-	-	-	-	-	-	2.6	-	-	-	-	-	-	0.1	0.2
Prostate	10	-	-	-	-	-	-	-	-	-	-	-	-	25.7	-	16.3	17.2	1.0	1.7
Testis	1	-	-	-	-	-	-	-	-	-	2.6	-	-	-	-	-	-	0.1	0.2
Other male genital organs	1	-	-	-	-	-	-	-	-	2.0	-	-	-	-	-	-	-	0.1	0.1
Kidney	1	-	0.7	-	-	-	-	-	-	-	-	-	-	-	-	-	-	0.1	0.1
Bladder	1	-	-	-	-	-	-	-	-	-	-	-	-	-	8.9	-	-	0.1	0.3
Brain, nervous system	1	-	-	0.8	-	-	-	-	-	-	-	-	-	-	-	-	-	0.1	0.1
Thyroid	1	-	-	-	-	1.1	-	-	-	-	-	-	-	-	-	-	-	0.1	0.1
Adrenal gland	1	-	-	-	-	-	-	1.5	-	-	-	-	-	-	-	-	-	0.1	0.1
Hodgkin disease	3	-	-	0.8	-	-	-	-	-	-	5.1	-	-	-	-	-	-	0.3	0.4
Non-Hodgkin lymphoma	5	-	-	-	-	-	2.5	-	-	-	-	3.0	-	5.1	-	8.1	-	0.5	0.7
Lymphoid leukaemia	1	-	-	-	-	-	-	-	-	2.0	2.6	3.0	8.9	-	-	-	5.7	0.1	0.1
Other unspecified	8	-	-	-	-	1.1	-	1.5	-	2.0	-	-	-	-	-	-	-	0.8	1.1
All sites	76	0.7	0.7	1.6	-	4.2	6.3	7.4	3.5	18.3	28.2	14.8	26.6	56.5	26.6	40.6	34.4	7.4	**10.9**

## Discussion

Our review of cancer in Kumasi is based on data collected with a reference population in mind in order to provide the basis for future estimation of the burden of cancers in Ghana and to assess the progress of cancer control programmes [1,2,19]. While a national PBCR may be ideal, the challenges to establishing such registries may include the lack of a uniform reporting system, the lack of a single national identification system, the multiple levels of independent healthcare delivery and the poor distribution of expertise in cancer diagnosis and treatment. Our use of multiple variables to reduce the incidence of multiple registrations is an example of actions that can be pursued to ensure that quality data is available for use in cancer prevention and control programmes.

Kumasi has largely been regarded as being conducive for the establishment of a PBCR because of the availability of laboratories and expertise to manage cancers. It also has fewer facilities offering oncology services allowing for cases to be easily identified and included in the registry database. Despite the challenges to cancer registration in developing countries [1,28], our review of the first dataset from a PBCR in Ghana indicates that some of these challenges can be overcome and good quality population-based data obtained to inform planning and cancer control activities.

Cancer in Ghana has evolved over the last few decades. While this may not necessarily be indicative of changes in disease pattern, one of the commonest cancers recorded in the late 1950’s was cancer of the Skin accounting for over a tenth of cancer cases recorded in Ghana [24]. Although skin cancers were rare in our dataset, we believe the observed high incidence in the late 1950s may be due to cases among white Ghanaian residents as Ghana was a British Colony at the time. Recent mortality reviews among cancer cases have shown lower rates of skin cancer-related mortality in Ghana [17].

The types of cancers recorded in Kumasi show some variation from studies on cancers in Ghana as well as other parts of Africa. There are also some consistencies observed. The high proportion of female cases is consistent with findings in the Gambia [5], Nigeria [30], Morocco [31] and from mortality records in Ghana [17]. Breast and cervical cancers were the leading cancers among females and this is also consistent with findings in other parts of Sub-Saharan Africa [5,7,30] although much lower rates were recorded in Kumasi compared with Ibadan and Abuja in Nigeria [30]. Liver cancer was the leading cancer among males in our review and is consistent with findings in the Gambia [5]. Prostate cancer, although the second leading cause of cancers in Kumasi among males, accounted for a lower proportion compared with 28% in Abuja and 23% in Ibadan [30]. Ovarian cancer as a proportion of female cancers was much higher in Kumasi (11.3%) compared with Nigeria where a proportion of 3% was recorded [30]. Lung cancers accounted for 1.6% for all cancers in our review and is consistent with findings in Nigeria (30) as Ghana and Nigeria share similar population characteristics. This may be due to the low prevalence of smoking [11,12] although the proportions are much lower compared with those recorded in other African countries as the Gambia (4%), Morocco (19%), [5,31].

### Limitations

Our review is limited in terms of coverage by virtue of the fact that not all possible data sources in the Kumasi metropolitan area were included in the data collection. However, we estimate that the majority of cases of cancers in Kumasi are seen in KATH as it is the only hospital in the region with the requisite human resource and logistics to manage cancer cases. We are also likely to have missed cases among residents who may have sought treatment elsewhere outside the Kumasi city. This though may be difficult to ascertain as there currently exists no national database on cases of diseases seen in health facilities. It is unlikely that this will be solved in the near future as challenges exist at the national level on the quality of data. These may have accounted for the low incidence recorded. Our ranking of cancer cases (Table 5) show some consistency with those recorded by GLOBOCAN. However our estimates are low and may be due to some of the factors highlighted. Despite this, minimal variations are seen in the leading sites of tumours occurring among both sexes in Ghana. GLOBOCAN estimates the leading cancers in descending order as Liver, Prostate, Non-Hodgkin Lymphoma, Colorectum and Lip and Oral Cavity for males. Among females, the leading cancers were Cervix, Breast, Liver, Ovary and Non-Hodgkin Lymphoma [32]. In the absence of any form of population-based data on cancers in Ghana, our data presents the best quality of population-based data yet, to be produced in Ghana.

**Table 5:** Top ten sites of cancers recorded in Kumasi (both sexes)

**Site**	**Frequency**	**Percentage**
Breast	61	24.1
Cervix	52	20.6
Ovary	20	7.9
Liver	16	6.3
Prostate	10	4.0
Endometrium	8	3.2
Stomach	5	2.0
Lung	4	1.6
Kidney	3	1.2
Urinary bladder	3	1.2
Others	71	28.1
Total	253	100.0

We may also have underestimated the incidence of breast cancer in Kumasi as a private hospital in Kumasi was not included in our dataset because of administrative challenges. However, being the only tumour seen at this clinic, the exclusion of data from this site can only affect the overall incidence of cancer in Kumasi and breast cancers in particular but not the ranking of cases of cancers in Kumasi.

## Conclusion

Population-based cancer registries are feasible in developing countries despite the challenges. Further strengthening of the cancer surveillance system in Kumasi in addition to the establishment of more PBCRs in Ghana is recommended in order to better estimate cancer incidence in Ghana and allow for evidence-based planning for cancer prevention and control.

## Abbreviations

PBCR: Population-based cancer registry; AFCRN: African Cancer Registry Network; KATH: Komfo Anokye Teaching Hospital; ASR: Age-standardised Incidence Rates; ICD-O3: International classification of diseases for oncology 3^rd^ edition; KsCR: Kumasi Cancer Registry; HPV: Human papilloma virus; HHV: Human herpes virus; HBCR: Hospital-based cancer registry.

## Competing interests

The authors declare no competing interests.

## Authors’ contributions

DOL, YAA, EOB, IKM, and BA conceived and designed the study. DOL, YAA, EOB, JD, NOF, RL-R, TOK, FKA, SBN, NAT, YE, KOB, JO and DA were responsible for data collection and abstraction. DOL, FKA, IKM and BA were responsible for data management. DOL, FKA, and YAA performed the statistical analysis. DOL and YAA wrote the manuscript with contributions from all authors. All authors read and approved the final manuscript.

## Authors’ information

DOL is a Public Health Specialist and manages the Kumasi Cancer Registry. He holds a Membership in Public Health at the Ghana College of Physicians and Surgeons. He is also the Head of the Public Health Unit of KATH. BA is a Consultant Radiation Oncologist and Director of the KsCR. He serves on the KsCR Advisory Board. YAA is a Fellow of the West African College of Physicians. He is currently a Senior Specialist Physician at KATH and serves on the Advisory Board of the KsCR. EOB is a Radiation Oncologist and is currently the Head of the Oncology Department of the KATH. NOF is a Biostatistician and the former Head of the Biostatistics Unit of KATH. He serves as a member of the KsCR Advisory Board. JD is a Paediatric Oncologist and a Fellow of the West African College of Physicians. He is a Lecturer in Paediatrics at the Department of Child Health of the Kwame Nkrumah University of Science and Technology, Kumasi, Ghana. FKA is a Statistician and a Registrar with the Kumasi Cancer Registry. He also manages data collected for the KsCR. TOK is a Consultant Obstetrician Gynaecologist and a lead Gynaecological Oncologist at the Department of Obstetrics and Gynaecology, KATH. SBN is a Paediatrician and a Fellow of the West African College of Physicians. He serves on the KsCR Advisory Board. NAT is a Consultant Pathologist and the Head of the Pathology Department of KATH. He is also a member of the KsCR Advisory Board. He is also a Lecturer in Pathology at the KNUST School of Medical Sciences. KYA is a Public Health Specialist and the Metropolitan Director of Health of the Ghana Health Service, Kumasi. KOB is a Specialist Obstetrician Gynaecologist and Head of the Obstetrics and Gynaecology Department and the Clinical Coordinator of the Kumasi South Regional Hospital. He is also the Clinical Coordinator at the Kumasi South Regional Hospital. RL-R is a Senior Specialist ENT Surgeon and runs the Head and Neck Oncology Clinic with the Department of ENT, KATH. She also serves on the KATH’s Head and Neck Tumour Board. DA is a Paediatrician and the Head of the Research and Development Unit of the Komfo Anokye Teaching hospital. He is also a Senior Lecturer in Paediatrics and the KbNUST School of Medical Sciences. JO is a Consultant General Surgeon and Lead Clinician of the Department of Surgery of the Komfo Anokye Teaching Hospital. IKM is a Fogarty Fellow with the University of Washington and has diverse experience on cancer registration in Sub-Saharan Africa.

## Pre-publication history

The pre-publication history for this paper can be accessed here:

http://www.biomedcentral.com/1471-2407/14/362/prepub
